# Mental health problems associated with idiopathic anaphylaxis

**DOI:** 10.1186/s13223-023-00824-0

**Published:** 2023-09-13

**Authors:** Logan S Gardner, Zihao Tan, David Brown, David Gillis, James G. Scott, Roger Prentice

**Affiliations:** 1 NSW Health Pathology, Department of Immunology, ICPMR, Westmead Hospital, Westmead, Sydney, Australia; 2 Royal Brisbane and Women’s Hospital, Brisbane, Australia; 3 Faculty of Medicine, University of Queensland, Brisbane, Australia; 4 Faculty of Medicine, University of Sydney, Sydney, NSW Australia; 5 Westmead Institute for Medical Research, University of Sydney, Sydney, NSW Australia; 6 Mental Health Programme, QIMR Berghofer Medical Research Institute, Herston, Qld Australia; 7 Metro North Mental Health Service, Herston, Qld Australia; 8 Children’s Health Research Centre, The University of Queensland, Brisbane, Australia

**Keywords:** Allergies, Psychiatry, Anxiety, Depression, Stress, Idiopathic anaphylaxis

## Abstract

**Background:**

Idiopathic Anaphylaxis (IA) is the most common anaphylactic syndrome in adults. Mental health problems associated with IA are not well recognised. We aimed to assess if patients diagnosed with IA were more likely to experience mental health problems compared to a normative Australian population. We additionally hypothesised that the number of anaphylactic episodes would correlate with symptoms of anxiety.

**Methods:**

A total of 34 patients with at least one episode of IA were recruited from an adult immunology clinic. Patients were recruited as part of a separate study evaluating alternative aetiologies in IA. Mental health problems were measured using the Depression, Anxiety and Stress Scale (DASS-21). An extension of the survey included questions specifically focused on the psychological impact of IA.

**Results:**

Compared to population norms, those with IA had significantly higher levels of mental health problems. Statistically significant DASS-21 scores were identified for depression 4.24 vs. 2.57 (p < 0.001), anxiety 4.76 vs. 1.74 (p < 0.012), stress 7.35 vs. 3.95 (p < 0.001) and total score 16.35 vs. 8.00 (p < 0.001). There was no association between two or more episodes of anaphylaxis and increased anxiety levels (β = 0.52, CI -2.59–3.62, p = 0.74).

**Conclusions:**

This is the first paper to demonstrate that patients living with idiopathic anaphylaxis are more symptomatic for mental illness than those in the community. Screening for mental illness and referral for psychological support should be undertaken in people with IA.

**Supplementary Information:**

The online version contains supplementary material available at 10.1186/s13223-023-00824-0.

## Introduction

Anaphylaxis is a potentially life-threatening hypersensitivity reaction with acute onset of a combination of urticaria, angioedema and end-organ dysfunction [1]. Cardiovascular, gastrointestinal and respiratory systems are commonly affected. The rapid onset of symptoms and the potentially life-threatening nature may increase the risk of mental health problems [2].

Allergies and anaphylaxis are increasing in incidence with related hospital admissions in the United Kingdom increasing by 615% between 1992 and 2015 [3]. Similarly, increases have been identified in Australia [4], New Zealand [5], and the United States [6]. It is estimated that up to 5% of the United States population has suffered anaphylaxis, although fatal outcomes occur in only 0.1% of patients presenting to emergency departments [7]. Despite the increasing incidence of anaphylaxis, the risk of mortality has steadily declined.

The aetiology of anaphylactic reactions is largely influenced by age, with food being the most common trigger in paediatric populations, while idiopathic anaphylaxis and drug reactions being the most common in adults [8].

Idiopathic anaphylaxis (IA) is a recognised entity with identical clinical characteristics to antigen mediated events. It was first described by Bacal, Patterson and Zeiss in 1978 in 11 patients with recurrent anaphylaxis without clear cause [9]. The diagnosis is one of exclusion made after patients have been thoroughly evaluated for potential immunogenic causes including foods, medications, insect stings and other conditions such as Mastocytosis [10]. Despite extensive investigation the aetiology of anaphylaxis remains elusive in 30–60% of adult cases [11].

The underlying pathophysiology of IA is unclear with multiple theories including a hidden and unidentified allergen, and fundamental abnormalities of the immune system including clonal mast cell disorders [12–14]. There are also suggestions that the condition may be related to female sex hormones given the higher incidence in females [13].

Regardless of the aetiology, anaphylaxis is an anxiety provoking event, with an impending sense of doom considered one of the identifiable symptoms [2]. It is reasonable to hypothesise that idiopathic anaphylaxis is associated with higher levels of mental health problems, given the uncertainty of when a further reaction may occur. In adults the effect of anaphylaxis has not received significant attention and to date, no studies have examined the association between IA and mental health problems.

The aim of this study was to measure mental health problems characterised by symptoms of depression, anxiety and stress in adults with idiopathic anaphylaxis. We hypothesised that compared to the general population, those with IA would have higher levels of mental health problems. Further, those who had two or more anaphylactic episodes would have higher levels of anxiety compared with those who had experienced a single episode.

## Methods

### Subjects and enrolment criteria

Subjects were recruited from the Royal Brisbane and Women’s Hospital, Brisbane, Australia between March 2019 to January 2020. The diagnosis of anaphylaxis was based on patients meeting the clinical criteria set forth by Sampson et al. [1]. In the majority of patients, a post event tryptase value was not available. Consenting patients with a diagnosis of anaphylaxis were approached to participate. Inclusion criteria were, confirmed anaphylaxis with no identified cause and age over 16 years. Patients were recruited as part of a study investigating the aetiology of IA. Those with a high suspicion of a specific aetiological antigen were excluded. Patients were identified as having IA by their treating Allergist and then enrolled into the study by a single physician who performed a detailed history and examination on each patient to further exclude an alternative diagnosis.

### Measures

A patient survey containing 36 questions was given to each participant which included the 21 item Depression, Anxiety, and Stress Scale (DASS-21) [15]. The DASS-21 was selected due to its ability to discriminate between anxiety and depression and the availability of normative scores for Australian adults. The scale is well validated with robust psychometric properties and commonly used for assessing mental health problems in Australian clinical settings [16]. It consists of three subscales which measure symptoms of depression, anxiety and stress. Each subscale is given a numerical score indicative of the severity of clinical symptoms.

Additional questions examining the impact of IA were also asked (Supplement Table 1). These focused on the impact of IA on the person’s health and broader functioning (e.g. leisure, work and travel), their access to health care and their concern about further episodes. Numerical questions were scored on a zero to ten visual analogue rating scale with associated descriptive nomenclature to aid the participant.

### Statistical methods

Mean total and sub-scale DASS-21 scores were compared with normative scores for Australian adults developed by Crawford et al. through an independent two-sample t-test using summary statistics [17]. Patients were dichotomised into those who had a single event (n = 10) and those who had two or more episodes (n = 24)) and a linear regression analysis was used to examine the anxiety subscale for the two groups.

Average scores for the 12 IA specific questions were reported as an overall mean result. Written answers were tabulated and later heuristically analysed.

## Results

A total of 34 patients that were recruited to the study and completed the survey. Baseline demographic and clinical data for patients can be found in Table 1. The majority of patients in this study were female 74% and only 3% of patients had received mental health care prior. Previous atopic conditions occurred in 55.9% of patients and drug or food allergies in 44.1% of patients.

**Table 1:** Patient Baseline Characteristics

Demographic and clinical characteristics at baseline	Result (n = 34)
Age, mean years +/- SD	41.6 +/- 17.5
Sex	
Male (%)	9 (26.5)
Female (%)	25 (73.5)
Previous treatment for mental illness (%)	3 (8.8)
Atopic History (%)	19 (55.9)
Previous Allergies (%)	15 (44.1)
Food	9 (26.5)
Medication	7 (20.6)
Insects	3 (8.8)
Number of Anaphylaxis events	
Total, mean +/- SD	7.6 +/- 14.3
Previous 6 months, mean +/- SD	2.5 +/- 8.5
Previous 2 months, mean +/- SD	1.0 +/- 2.7
Following food ingestion	9 (26.5)

DASS-21 scores in the clinical population and Australian normative data are presented in Table 2. The mean total DASS-21 score for IA patients was 16.35 compared to 8.30 in normative patients (P < 0.001). Scores on all three subscales of the DASS were significantly greater in the IA clinical sample than the Australian population (Table 2).

**Table 2:** DASS-21 scores in Idiopathic Anaphylaxis group compared with the general Australian population

	Idiopathic anaphylaxis *(n = 34)*	General population* *(n = 497)*	Difference 95% CI	P value
Depression m(SD)	4.24 (4.46)	2.57 (3.68)	1.67 0.37–2.97	< 0.001
Anxiety m(SD)	4.76 (3.99)	1.74 (2.78)	3.02 2.02–4.02	0.012
Stress m(SD)	7.35 (4.85)	3.99 (4.24)	3.36 1.87–4.85	< 0.001
Total m(SD)	16.35 (12.25)	8.30 (9.83)	8.05 4.57–11.53	< 0.001

*Source: Crawford, 2011

The DASS severity rating for each patient is shown in Table 3. This demonstrates that a significant proportion of patients with IA experienced comorbid mental health problems with elevated symptoms of depression (29.4%), anxiety (53.0%), or stress (35.2%).

**Table 3:** DASS Severity rating for the clinical population

**Depression**	**Number (%)**
Normal	24 (70.6)
Mild	2 (5.9)
Moderate	5 (14.7)
Severe	1 (2.9)
Extremely severe	2 (5.9)
**Anxiety**	**Number (%)**
Normal	16 (47.1)
Mild	4 (11.8)
Moderate	8 (23.5)
Severe	2 (5.9)
Extremely severe	4 (11.8)
**Stress**	**Number (%)**
Normal	22 (64.7)
Mild	6 (17.6)
Moderate	1 (2.9)
Severe	3 (8.8)
Extremely severe	2 (5.9)

There was no association between two or more episodes of IA and increased anxiety (β = 0.52, CI -2.59–3.62, p = 0.74).

Assessment of the impact of IA on patients’ daily lives are summarised in Table 4 that is divided into four score ranges to aid interpretation. 73% of participants had significant concerns regarding further episodes regardless of whether they had multiple episodes or had not experienced an episode in the last six months. This concern would occasionally prevent them from engaging in activities they enjoy in over 44% of patients and would frequently prevent them from partaking in 15% of patients. A total of four patients stated they were no longer able to work due to their concern over further events. In remaining patients there was minimal limitation with 68% describing no effect.

**Table 4:** Idiopathic Anaphylaxis specific questions and visual analogue score results

Question	Average (SD) [Description*]	Score 0–2 (%)	Score 3–5 (%)	Score 6–8 (%)	Score 9–10 (%)
1. In general, how would you rate your health prior to the onset of symptoms of idiopathic anaphylaxis? *(Range: Poor - Very Good)*	6.7 (2.5) [Good]	11.8	8.8	55.9	23.5
2. In general how would you rate your health at present? *(Range: Poor - Very Good)*	6.2 (2.3) [Good]	5.9	38.2	41.2	14.7
3. Are you concerned about further episodes? *(Range: Not at all - Very)*	7.5 (2.6) [Moderate/Very]	5.9	20.6	26.5	47.1
4. Does your concern over further episodes prevent you from undertaking activities that you would enjoy? *(Range: Never - Frequent)*	5.1 (3.3) [Occasional]	29.4	26.5	29.4	14.7
5. Does your concern over further episodes prevent you from working? *(Range: Never - Frequent)*	2.4 (3.5) [Rarely]	67.6	11.8	8.8	11.8
6. Has your diagnosis restricted your diet? *(Range: Not at all - Very)*	4.1 (3.6) [Moderately]	41.2	23.5	17.6	17.6
7. Has your diagnosis affected your ability to travel? *(Range: Not at all - Very)*	3.2 (3.4) [Not at all /Moderately]	52.9	26.5	5.9	14.7
8. How well do you understand your condition? *(Range: Poor - Very Good)*	5.5 (2.9) [Good]	14.7	32.4	38.2	14.7
9. How do you think other people view your condition? *(Range: Life threatening - Not a real disease)*	4.2 (2.6) [Inconvenience]	23.5	47.1	20.6	8.8
10. How do you view your condition? *(Range: Life threatening - Not a real disease)*	3.5 (2.3) [Serious]	29.4	58.8	8.8	2.9
11. How well do you think your concerns have been addressed? *(Range: Poor - Excellent)*	7.1 (2.4) [Very Good]	0.0	29.4	38.2	32.4

* Correlates to the approximate guide descriptions provided on each visual analogue scale question

Patients described IA as having a moderate effect on their diet and less significant effect on their ability to travel, with 15% of participants modifying travel requirements. Patients generally considered themselves to be in good health before and after the diagnosis of IA and thought they had received good treatment from their Immunologist.

While patients in general self-regarded their condition as serious, they believed that the general population perceived their condition as more of an inconvenience. All patients had seen their General Practitioner within the last 2 months and 17% of patients had seen another specialist for their symptoms before presenting to an immunologist.

Patients were given the opportunity to provide feedback on specific concerns regarding IA. Themes identified included a fear of further events and that additional events may be more severe and life-threatening. Other common themes were the fear of the unknown, given that an unidentified precipitant could not be avoided, or events predicted. This led to several patients stating they have avoided taking holidays or traveling outside the reach of local hospitals.

Another finding were negative experiences with emergency medicine encounters. One patient stated, “I am worried that the local hospital does not understand or want to understand my condition. I find that going to hospital affects my mental health and I would rather risk my life and stay at home with a serious attack than face the emergency department”.

## Discussion

Previous assessments of the mental health symptoms associated with anaphylaxis and allergies have focused predominately on paediatric and adolescent populations and their parents rather than in adults [18–20]. These studies have demonstrated poorer quality of life and increased levels of anxiety and depression [20–22]. This study shows, for the first-time, that adult patients with IA have a higher burden of mental health problems than the general population.

Patients with idiopathic anaphylaxis face unique challenges. They are unable to be provided with an identifiable precipitant to avoid, making further potentially life-threatening reactions inherently unpredictable [13]. To our knowledge only four studies have addressed the mental health problems associated with anaphylaxis in adult populations [17, 23–25].

The first study reported associated mental health problems with mast cell disorders in 420 patients where 41% of patients reported the condition has had an extreme effect on their lives, 61% experienced anxiety and 49% reported depression [24]. IA is theorised to exist on a continuum with other mast cell disorders such as Mast Cell Activation Syndrome. This study utilised self-reported anxiety and not a validated assessment tool.

Another study using a semi-structured interview in 12 patients with adult onset anaphylaxis by Walklet et al., identified the control of triggers as core to the patients’ ability to cope and manage with the concept of further episodes of anaphylaxis [25]. Additionally, they noted that in patients where a trigger was not identified they were subjectively more significantly affected. Our study also identified the loss of autonomy and control as a significant stressor for patients with idiopathic anaphylaxis.

A third study by Baiardini et al., assessed mental health and wellbeing in adult patients that had survived anaphylaxis secondary to medications, provided a quantitative result by utilising two validated scores, the Drug Hypersensitivity Quality-of-Life Questionnaire, and the Psychological General Well-Being Index [23]. Patients exhibited significantly reduced health-related quality of life scores and increased distress compared to a community population. Our study provides quantitative evidence to demonstrate that patients with IA had higher symptom scores for depression, anxiety, and stress with most participants having mental health symptoms.

A previous meta-analysis has identified a bidirectional relationship between atopic disorders and mental ill-health with each affecting the prognosis of the other [26]. This interplay is thought to include a combination of behavioural and socioeconomic factors, though direct physiological pathways have been considered [26]. It has been theorised that inflammatory cytokines and neuropeptides released in atopic disorders influence the hypothalamic-pituitary-adrenal axis and transmission within the peripheral nervous system [26]. Direct effects on the central nervous system have been observed in allergen exposed rats with physical and signalling changes in the anxiety related brain regions of the medial prefrontal cortex and amygdala [27].

Furthermore the experience of an anaphylactic event linked to an unknown and therefore unmodifiable trigger could understandably induce a phobic response in individuals with IA. Consistent with the distress associated with anaphylaxis, a cross-sectional survey by Chung et al. of adult patients with anaphylaxis identified that 12% met the diagnostic criteria for post-traumatic stress disorder and a significantly higher proportion of other psychiatric comorbidities compared to a control population [17].

The limitations of this study include the small sample size recruited from a single site in a high-income country. The study is likely to have been underpowered to determine with confidence if there was no association between multiple episodes of anaphylaxis and increased anxiety. Given patients were enrolled after at least one episode of anaphylaxis the study is limited in its ability to attribute causation. Somatoform events are a recognised alternative diagnosis in recurrent anaphylaxis episodes [28], though such patients were not included in this study. A further limitation was in the diagnosis of anaphylaxis which was based on personal accounts and emergency physician descriptions. Therefore, it is possible that a small subset of these patients did not experience a true anaphylactic episode. The mental health problems were assessed by a symptom measure rather than a diagnostic clinical interview. There may have been a selection bias in relation to those who chose to participate. We hypothesise that participants were more functional and better educated and therefore the results may be limited in their generalisability. However, the study’s strengths include the well validated measure for assessing mental health problems, the research collaboration between the specialties in psychiatry and immunology and the combining of quantitative and some qualitative data of the psychosocial impact of IA on patients.

This study provides clear evidence of the increased risk of unrecognised mental health problems in this clinical population. It demonstrated that the majority of patients with idiopathic anaphylaxis also experience mental health symptoms, although only a small minority (8.8%) reported having a coexisting mental health diagnosis. This suggests that people with IA are not receiving optimal mental health care. Fortuitously, most are only experiencing a “mild” level of emotional problems, and these could most likely be addressed by mental health education and some easy to implement psychological strategies and lifestyle changes such as regular exercise, improving sleep hygiene, mindfulness, relaxation techniques and restructuring of unhelpful cognitions [29]. The integration of psychological interventions in immunology clinics through online resources may serve the mental health needs of the majority of these patients. Identification of the small proportion of people with high levels of mental health symptoms and selective referral to mental health clinicians would also be beneficial for holistic health care delivery.

In summary idiopathic anaphylaxis is a serious disorder with morbidity that encompasses both the physiological aspects of anaphylaxis and associated mental health problems. Attention to both the physical and psychological needs of these patients is likely to lead to improved health outcomes. Further research is needed to determine the best model of service to meet the health needs of people living with IA.

### Electronic Supplementary Material

Below is the link to the electronic supplementary material.

**Supplementary Material 1**: Table 1: Patient Disorder Perspective

## Data Availability

The authors confirm that the data supporting the findings of this study are available within the article and its supplementary materials.

## Abbreviations

DASS: Depression, Anxiety and Stress Scale;
IA: Idiopathic anaphylaxis
