# Supplementary material for: Mental health problems associated with idiopathic anaphylaxis
Source: Allergy Asthma Clin Immunol. 2023 Sep 13;19:84. doi: 10.1186/s13223-023-00824-0 (PMC10500772; doi:10.1186/s13223-023-00824-0)
Supplement: Supplementary file 1 — Supplementary Material 1: Table 1: Patient Disorder Perspective [file 13223_2023_824_MOESM1_ESM.docx]

**Supplementary Table 1: Patient Disorder Perspective**

| Question | Rating (scale, description) |
| --- | --- |
| 1. In general, how would you rate your health prior to the onset of symptoms of idiopathic anaphylaxis? | Scale 0 - 10, Poor - Very Good |
| 2. In general how would you rate your health at present? | Scale 0 - 10, Poor - Very Good |
| 3. Are you concerned about further episodes? | Scale 0 - 10, Not At All - Very |
| 4. Does your concern over further episodes prevent you from undertaking activities that you would enjoy? | Scale 0 - 10, Never - Frequent |
| 5. Does your concern over further episodes prevent you from working? | Scale 0 - 10, Never - Frequent |
| 6. Has your diagnosis restricted your diet? | Scale 0 - 10, Not At All - Very |
| 7. Has your diagnosis affected your ability to travel? | Scale 0 - 10, Not At All - Very |
| 8. How well do you understand your condition? | Scale 0 - 10, Poor - Very Good |
| 9. How do you think other people view your condition? | Scale 0 - 10, Life Threatening - Not a real disease |
| 10. How do you view your condition? | Scale 0 - 10, Life Threatening - Not a real disease |
| 11. How well do you think your concerns have been addressed? | Scale 0 - 10, Poor - Excellent |
| Questions 12 to 32 – DASS-21 | Scale 0 - 4 |
| 33. When was the last time you saw your GP? | Written |
| 34. Which other specialties have you seen for your condition? | Written |
| 35. How long was it from symptom onset to diagnosis | Written |
| 36. What are your current concerns regarding your diagnosis of Idiopathic Anaphylaxis | Written |
